# The identification and genetic characteristics of the Orf virus strain (ORFV-CL24) isolated from Jilin province, China

**DOI:** 10.3389/fmicb.2025.1658326

**Published:** 2025-09-24

**Authors:** Lijun Lv, Fei Gao, Jiyu Guan, Yiran Sun, Ran Zhang, Zi Li, Yungang Lan, Feng Gao, Wenqi He, Kui Zhao

**Affiliations:** ^1^State Key Laboratory for Diagnosis and Treatment of Severe Zoonotic Infectious Diseases, Key Laboratory for Zoonosis Research of the Ministry of Education, Institute of Zoonosis and College of Veterinary Medicine, Jilin University, Changchun, China; ^2^Department of Laboratory Animals, College of Animal Science, Jilin University, Changchun, China

**Keywords:** ORFV, complete genome, genetic characteristics, phylogenetic analysis, genetic evolution

## Abstract

Orf virus (ORFV), the prototype species of the *Parapoxvirus* genus within the *Poxviridae* family (subfamily *Chordopoxvirinae*), is a global pathogen infecting sheep, goats, and other ruminants, with zoonotic potential for humans. In this study, an outbreak of ORFV infection occurred in a sheep flock in Changling County, Jilin province, China, causing papules, pustules, and crusting lesions on the lips and eyelids. Typical parapoxvirus particles were observed using electron microscopy, and a wild ORFV strain was isolated, characterized, and designated as ORFV-CL24. To clarify the epidemiological and genomic characteristics of ORFV in the region, we completed its whole-genome sequencing (GenBank accession number: PV126639). Genome analysis revealed that ORFV-CL24 shares a conserved structure with other isolates available in GenBank, which possess a complete genomic sequence of 138,500 bp of dsDNA harboring 131 putative open reading frames (ORFs) flanked by inverted terminal repeat (ITR) regions of 3,264 bp at both termini. Additionally, the genome exhibited high GC-content (63.3%), indicating its key role in DNA stability. Phylogenetic analysis placed the wild strain within a subclade with the attenuated ORFV strain D1701, implying a putative common ancestor or epidemiological linkages. Further analysis of B2L (ORFV 011) and E3L (ORFV 020) genes further revealed genetic diversity and evolutionary patterns. Notably, despite phylogenetic relatedness, specific mutations in ORF020 further distinguished ORFV-CL24 from D1701, reflecting stepwise mutation accumulation during host adaptation. In conclusion, our results provided valuable genetic insights into ORFV-CL24, which contributed to a better understanding of its evolution, biological properties, and endemic trends in China.

## Introduction

Orf, also known as contagious ecthyma (CE) or contagious pustular dermatitis, is an acute, highly contagious zoonotic disease caused by ORFV, which is widely distributed in countries and regions with developed sheep industries (Lawan et al., 2021). The disease predominantly affects sheep, goats, and, to a lesser extent, humans and other animals such as muskoxen or camels (Tryland et al., 2018; Al-Bayati, 2022; Abu Ghazaleh et al., 2023). Characteristic clinical signs include ulcerative lesions and papulopustular eruptions on the skin of the lips, in and around the mouth, and the eyelids, accompanied by systemic symptoms in severe cases, such as secondary infections and polymicrobial coinfections (Efridi et al., 2025). In healthy humans, it usually presents as sores or nodules on the hands or arms (Centers for Disease Control and Prevention (CDC), 2012; Kassa, 2021; Salvi et al., 2024). Although Orf is recognized as a typical self-limited disease, often resolving spontaneously within 3–6 weeks, it has emerged as a significant zoonotic threat with escalating annual mortality rates, primarily due to the lack of effective antiviral treatment for ORFV infection (Hosamani et al., 2009; Spyrou and Valiakos, 2015).

ORFV belongs to the *Parapoxvirus* genus in the *Poxviridae* family (Coradduzza et al., 2024). This genus currently encompasses four recognized species according to the International Committee on Taxonomy of Viruses (ICTV): ORFV, pseudocowpox (PCPV), bovine papular stomatitis virus (BPSV), and red deerpox virus (RDPV) (Friederichs et al., 2014), which can be distinguished from other poxviruses by several distinctive features: ovoid virion morphology, relatively small size (approximately 260 × 160 nm), exceptionally high guanine–cytosine G + C content (63–64%), and a unique spiral (criss-cross) surface pattern observed by electron microscopy (Gelaye et al., 2016). ORFV is a complex, linear, double-stranded DNA virus with a genomic length of approximately 138 kbp, which harbors approximately 132 genes involved in various important biological characteristics, such as viral replication, morphogenesis, and pathogenesis (Fleming et al., 2015). Similar to other poxviruses, the core region of the ORFV genome is highly conserved, which encodes proteins indispensable for the virus’s life cycle, including replication, assembly, and release (Haig and Mercer, 1998; Yao et al., 2020). In contrast, the terminal regions are involved in viral invasion, immune evasion, and host-range determination. In addition, the ORFV genome is flanked by two inverted terminal repeats (ITRs) of approximately 3 kbp, which are covalently closed by 100 bp hairpin loops, thus completing the unique genomic architecture of ORFV (Li et al., 2015). The unique genome arrangement not only contributes to the genetic stability of the viral genome, but also aids the replication, packing, and its overall evolutionary trajectory.

Over the past few years, an increase in the occurrence and prevalence of ORFV infections has been reported in flocks all over the world, resulting in serious economic losses. Thus, the increasing investigations have been driven by the growing prevalence of ORFV infections. Due to the genetic variability among strains and the sophisticated immune evasion mechanisms, it is difficult to develop improved diagnostics, effective therapeutics, and control measures for ORFV infections, which highlights the urgent need for comprehensive genomic characterization to elucidate pathogenic molecular determinants (Haig, 2006; AlDaif and Fleming, 2025). Thus far, only a few complete genome sequences have been publicly reported, hence largely hindering our understanding of epidemiology, biology, and pathogenesis of ORFV in flocks. In this present study, to better understand the characteristics of the ORFV epidemic strain, we successfully completed the genomic sequences of the ORFV-CL24 strain, isolated from naturally infected sheep in Jilin province, northeastern China. Furthermore, the phylogenetic analysis was performed on the complete genome sequences and individual genes, including B2L and E3L, to determine the evolutionary relationship among ORFV-CL24 and other ORFV strains. The results indicated that the ORFV-CL24 strain exhibited the closest evolutionary relationship with the attenuated ORFV strain D1701, implying a putative common ancestor or sharing a common ancestor with the pre-attenuated D1701. This study provided key evidence for deeply understanding the genetic diversity and molecular evolution patterns of the ORFV epidemic strain and the endemic situation of Orf in China.

## Materials and methods

### Tissue sampling

A natural outbreak of Orf on a sheep breeding farm occurred in Changling County of Jilin province in northeastern China in 2024. The affected animals were suspected to be infected with ORFV, based on characteristic clinical signs, such as papules, pustules, and crusting lesions localized to the lips and eyelids. In accordance with standard protocols, the affected sheep was euthanized humanely to obtain tissue samples for comprehensive diagnostic evaluation. Fresh tissue specimens for ORFV isolation were aseptically collected from active vesicular lesions and scab tissues in both perioral and periocular regions, which were promptly preserved at −80 °C to maintain viral viability. Simultaneously, representative lesional tissues were carefully excised and fixed in 10% neutral-buffered formalin under controlled conditions to ensure optimal tissue preservation for subsequent histopathological assessment.

### Histopathology investigation

The formalin-fixed tissue samples were embedded in paraffin wax blocks, then they were cut into 4 μm-thick slices and stained with hematoxylin and eosin (H&E). The microscopic assessment under light microscopy was then performed to visualize the pathological aspects of the analyzed tissues, and high-resolution pictures with 20 × magnification were taken.

### Virus isolation

The scab tissues from the affected area of lambs with typical pathological changes consistent with ORFV infection were collected and homogenized in 0.01 M phosphate-buffered saline (PBS). The tissue homogenate was subjected to three cycles of freeze and thaw, followed by centrifugation at 5000 × *g* for 10 min at 4 °C. The clarified supernatant fluids were collected and used for virus isolation. Briefly, the supernatant filtered through a 0.45 μm filter was inoculated onto confluent monolayers of primary ovine fetal turbinate (OFTu) cells maintained in Dulbecco’s Modified Eagle Medium (DMEM) supplemented with 2% fetal bovine serum (FBS) in an incubator at 37 °C with 5% CO_2_, with the uninfected cells serving as a negative control. The cytopathic effect (CPE) was observed daily under an inverted microscope. After five serial passages, the inoculated cells showed the characteristic morphological changes, including cell shrinkage, rounding, and detachment. Then, the resulting supernatant was collected as the P0 stock and passaged one additional time on OFTu cells. Thus, the ORFV-CL24 strain (GenBank accession number: PV126639) was successfully isolated using OFTu cells from the naturally infected sheep in Changling County of Jilin province in 2024 and maintained in our laboratory.

### DNA extraction and PCR

Viral DNA was extracted from 200 μL of viral stocks using the TIANamp DNA extraction kit (Tiangen Biotech, Beijing, China) according to the manufacturer’s instructions. The polymerase chain reaction (PCR) was conducted to ascertain the presence of ORFV. Three pairs of specific primers targeting the ORFV 011, ORFV 020, and ORFV 059 were designed using the software Primer Premier 5. The sequences of the primers are as follows: ORFV 011-Fw: 5′-TTTCCACTCGGTGATGATTACGC-3′, and ORFV 011-Rv: 5′-CGAACTTCCACCTCAACCACTCC-3′, ORFV 020-Fw: 5′-GCTGATGCCGCAGTTGTTGA-3′, and ORFV 020-Rv: 5′-CCGCCTCGTTGGTTCGTAGA-3′, ORFV 059-Fw: 5′- CCAGCCGTTCGTCCTCATC-3′, and ORFV 059-Rv: 5′-CAAGGCGGTGGAATGGAAA-3′. PCR amplification was carried out with initial denaturation at 98 °C for 2 min, followed by 35 cycles of 98 °C for 15 s, 60 °C for 30 s, and 72 °C for 5 s, with a final extension at 72 °C for 5 min. The PCR products were analyzed on 1% agarose gel electrophoresis.

### Electron microscopy observation

To verify the presence of viral particles in the cell culture supernatants, the virions were detected using transmission electron microscopy. After removing dead cells and cell debris by low-speed centrifugation, the supernatants were collected and ultracentrifuged at 20,000 r/min for 90 min. Then, the pellet was resuspended in 50 μL of ice-cold PBS and negatively stained with 2% phosphotungstic acid. Subsequently, the morphology structure of virus particles was examined under a transmission electron microscope (TEM) at appropriate magnifications.

### Detection of virus antigens by western blotting

Viral antigens in CPE-positive OFTu cells were investigated using Western blotting. In brief, cells were lysed in RIPA buffer supplemented with protease inhibitors. Total protein extracts were separated by 12% SDS-PAGE and transferred to a polyvinylidene fluoride (PVDF) membrane. After blocking with 5% skim milk in TBST (Tris-buffered saline with 0.1% Tween-20), the membranes were incubated overnight with primary antibodies anti-ORFV (1:1000; Santa Cruz Biotechnology, Texas, USA) or anti-GAPDH (1:1000; Proteintech Group, IL, USA) at 4 °C. The membranes were then incubated with HRP-conjugated secondary antibodies in TBST for 1 h at 37 °C. Finally, the protein signals were detected by enhanced chemiluminescence (ECL) substrate (Biosharp, Beijing, China) and visualized using a chemiluminescence imaging system (Tanon, Shanghai, China).

### Immunofluorescent staining and titration of virus infectivity

The infectious viral particles were detected in ORFV-CL24-infected cells by indirect immunofluorescence assay (IFA). Briefly, OFTu cells cultured on sterile coverslips in 12-well plates were infected with ORFV-CL24 at a multiplicity of infection (MOI) of 1. When extensive CPE was observed, cells were fixed with 4% paraformaldehyde for 30 min at room temperature (RT), permeabilized with 0.1% Triton X-100 in PBS for 10 min at RT, and blocked with 5% skim milk in PBS for 1 h at 37 °C. After incubation with anti-ORFV antibody (1:500) overnight at 4 °C, and Alexa Fluor 488-conjugated secondary antibody (Cell Signaling Technology, MA, USA) in the dark for 1 h at 37 °C. Coverslips were washed and mounted using DAPI-containing antifade medium. Fluorescent images were acquired by a laser scanning confocal microscope (Olympus, Japan).

Viral titer was determined by 50% tissue culture infectious dose (TCID₅₀) endpoint assay and plaque assays. For the TCID₅₀ assay, 10-fold serial dilutions of virus supernatants (10^−1^ to 10^−9^) were inoculated onto confluent monolayers of OFTu cells in 96-well plates (100 μL/well), while control wells received medium only. After 1 h of adsorption at 37 °C, the inoculum was replaced with DMEM with 2% FBS and incubated for 7 days. The cells were monitored daily for CPE using an inverted phase-contrast microscope. Infectivity titers (TCID_50_/0.1 mL) were then calculated according to the Reed–Muench statistical method. For plaque assay, viral supernatants were serially diluted 10-fold in DMEM to achieve final dilutions of 10^−4^. When OFTu cells reached approximately 90% confluency in 12-well plates, 300 μL of diluted virus supernatant was inoculated at a volume of 300 μL per well in triplicate and then incubated at 37 °C with 5% CO₂ for 1 h to allow viral adsorption. Cells were overlaid with 1% low-melting-point agarose in MEM with 2% FBS and incubated for 3–5 days. Visible plaques were counted manually following crystal violet staining. Viral titers were calculated and expressed as plaque-forming units per mL (PFU/mL) as follows:


PFU/mL=(plaque number)×(dilution factor)^(−1)×(inoculum volume)^(−1).

### DNA sequencing and genome assembly

Viral genomic DNA was extracted from CPE-positive supernatants using a DNA extraction kit (Tiangen Biotech, Beijing, China), and the quality was assessed by 1% agarose gel electrophoresis. DNA was sheared to 200–400 bp fragments by ultrasonication, followed by end repair, adenylation, adapter ligation, and PCR amplification. The prepared library was purified with magnetic beads, quantified, and size-selected prior to 300 bp paired-end sequencing on the Illumina NovaSeq 6,000 platform (Sangon Biotech, Shanghai, China). Raw sequencing reads were processed with Trimmomatic (v0.36) to remove adapter sequences and low-quality bases. *De novo* assembly was performed using SPAdes (v3.15) with subsequent gap closure by GapCloser (v1.11). Gene prediction was conducted with GeneMarkS (v4.1), followed by functional annotation against both COG (Clusters of Orthologous Groups) and KEGG (Kyoto Encyclopedia of Genes and Genomes) databases. The complete ORFV-CL24 genome sequence has been deposited in GenBank under accession number PV126639.

Genome-wide characterization revealed that the genomic organization of ORFV-CL24 included a central coding region flanked by inverted terminal repeats (ITRs). Comparative sequence analysis of ORFV ITR was performed using DNAMAN (v7.02) and Clustal Omega^1^ (Madeira et al., 2024), with specific annotation of conserved features of the BamHI restriction site (5’-GGATCC-3′) and telomere resolution motif (5′-ATTTTTT-N8-TAAAT-3′) using SnapGene (v6.2.1)^2^. Genome visualization and annotation were performed with Proksee^3^ (Grant et al., 2023) to analyze coding sequence (CDS) orientation, GC content distribution, and GC skew patterns, supplemented by structural characterization by using Geneious Prime (v11.0.18)^4^.

### Phylogenetic analysis

Phylogenomic analysis of the ORFV-CL24 whole-genome sequence was performed through MAFFT-based alignment (v7.490) (Nakamura et al., 2018), TrimAI-mediated sequence trimming (v1.5.0) (Capella-Gutiérrez et al., 2009), and maximum-likelihood phylogenetic reconstruction using IQ-TREE 2 (v2.4.0) (Minh et al., 2020) with 1,000 ultrafast bootstrap replicates for branch support assessment. Specific investigations of ORFV 011 (Zhang et al., 2014) and ORFV 020 (Tseng et al., 2015) gene sequences incorporated multiple sequence alignment via Clustal Omega, phylogenetic reconstruction using the neighbor-joining algorithm, and comprehensive visualization of sequence conservation patterns through R (v4.4.2)-based heatmap analysis and detailed inspection using Jalview (v2.11.4.1) (Waterhouse et al., 2009).

## Results

### Gross pathological changes

**Figure 1:**
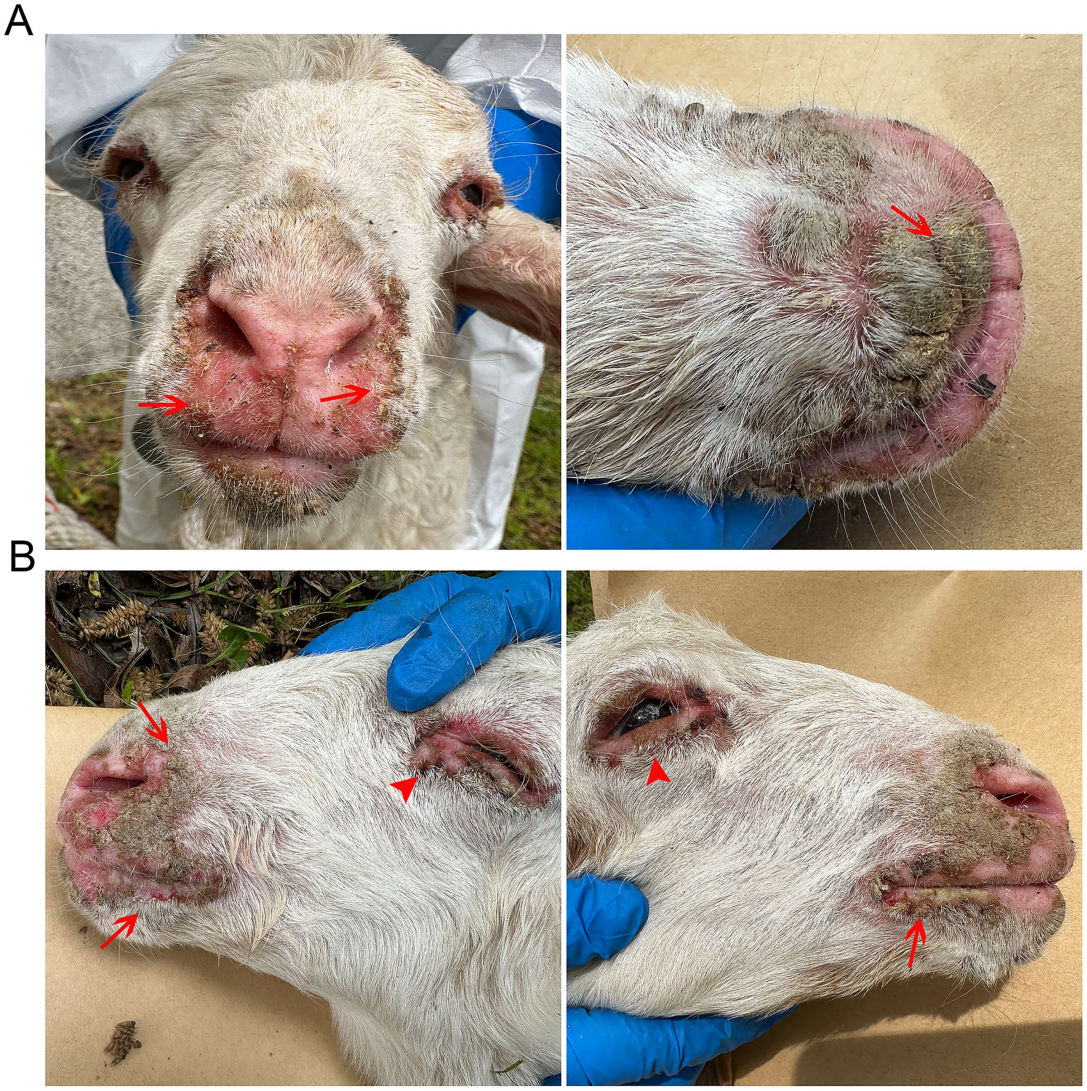
Oral-labial and eyelid lesions in ORFV-infected sheep. **(A)** Multiple nodule lesions with ulcers and crusts were shown on the lips, surrounded by erythematous and swollen skin. **(B)** Characteristic nodules with ulcers and mild hemorrhage were observed on the lips and the margins of the eyelids.

The natural outbreak of ORFV infection investigated in this study occurred in July 2024. Affected sheep displayed the hallmark clinical manifestations of ORFV infection that included multifocal vesicular-papular eruptions progressing to ulcerative lesions, and nodular proliferations with associated hemorrhagic foci predominantly localized to perioral and periocular regions (Figure 1). These gross pathological changes were strictly limited to the lips, eyelids, and nasal cutaneous and mucocutaneous sites, with comprehensive necropsy examination showing the absence of discernible lesions in any internal organs.

### Histopathological examination

**Figure 2:**
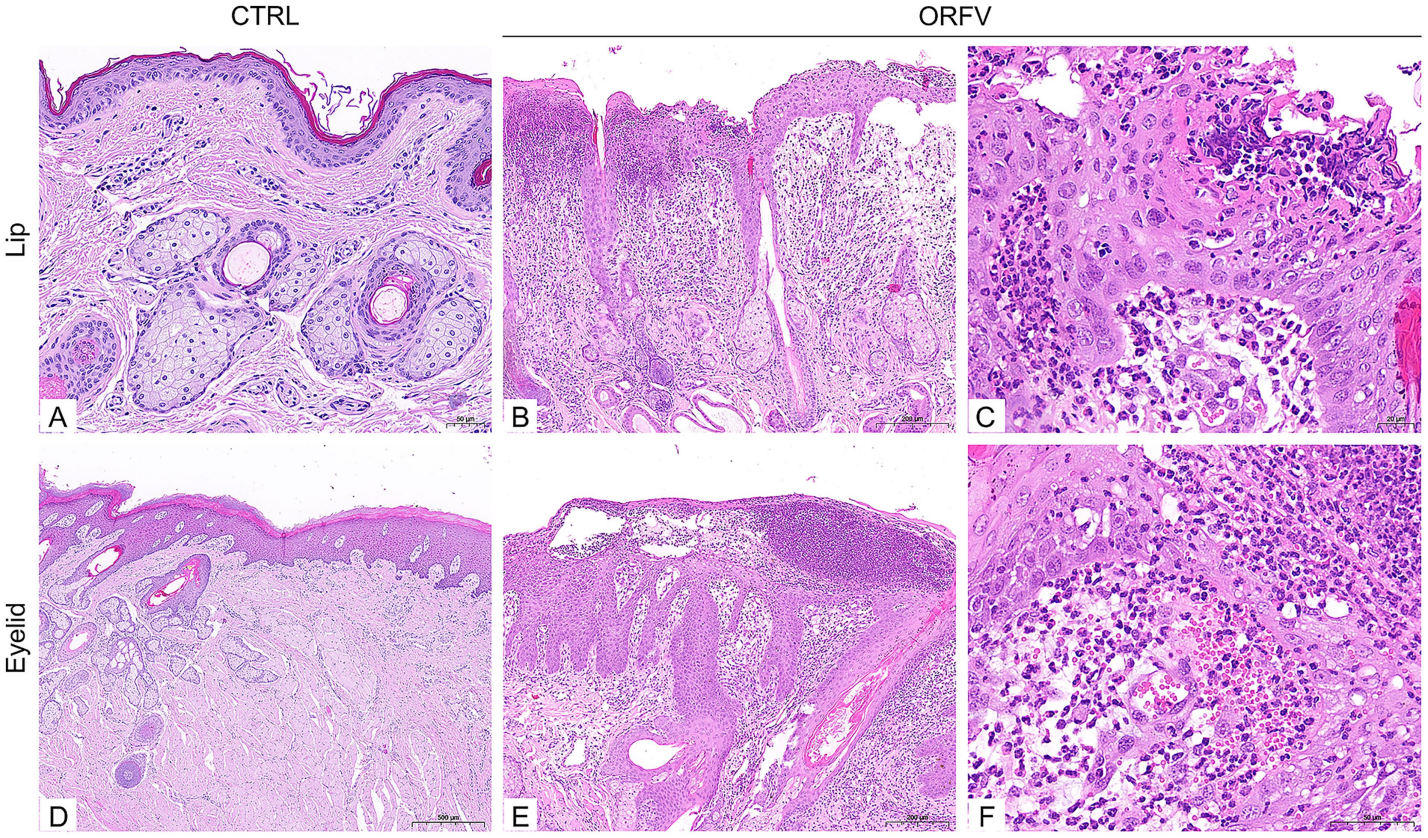
Histopathological features in the collected lip and periocular tissues of affected sheep (H&E-stained sections). **(A,D)** The morphology of normal tissue structures, including lips **(A)** and the margins of eyelids **(D)**. Scale bars: 50 μm **(A)**; 500 μm **(D)**. **(B,C)** The pathological changes in the lip tissues of affected sheep show marked epidermal hyperplasia with orthokeratotic hyperkeratosis and acanthosis **(B)**, karyorrhexis in spinous cells of the stratum spinosum, and inflammatory infiltration **(C)**. Scale bars: 200 μm **(B)**; 20 μm **(C)**. **(E,F)** Thickening of the spinous cell layer **(E)**, swelling, vacuolation, and karyorrhexis in spinous cells of the stratum spinosum, and predominantly neutrophilic, inflammatory infiltration were observed in the eyelids **(F)**. Scale bars: 2S00 μm **(E)**; F: 50 μm **(F)**. Romal edema with dense inflammatory infiltration. Scale bars: E: 200 μm; F: 50 μm.

Histopathological examination of H&E-stained sections involving the oral and periocular tissues of affected sheep revealed distinct pathological changes, including marked epidermal hyperplasia with orthokeratotic hyperkeratosis, acanthosis, and overlying hyperkeratosis and parakeratosis (Figures 2B,E). In addition, thickening of the spinous cell layer, swelling, vacuolation, and karyorrhexis in spinous cells of the stratum spinosum, and predominantly neutrophilic, inflammatory infiltration with microabscess formation and vascular dilation were observed (Figures 2C,F). On the other hand, no evident change was observed in the control group (Figures 2A,D).

### Virus isolation and identification

**Figure 3:**
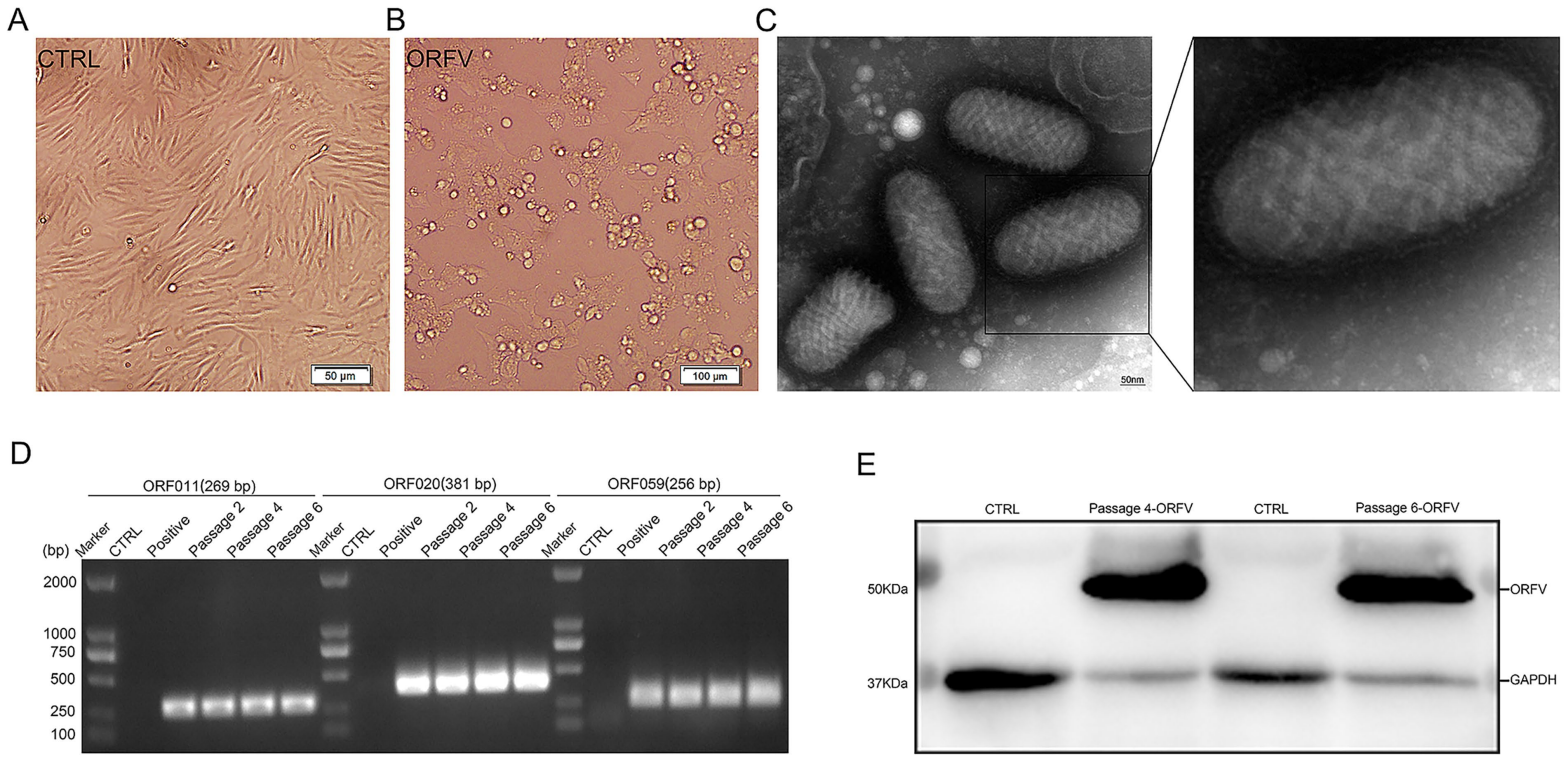
Isolation and identification of ORFV-CL24 strain by PCR, TEM observation, and Western blotting, respectively. **(A)** Normal control cells. Scale bar: 50 μm. **(B)** OFTu cells inoculated with the supernatants of the collected scab specimens displayed a typical CPE of ORFV infection characterized by cell rounding and detachment. Scale bars: 100 μm. **(C)** Typical enveloped, ovoid-shaped virions with the characteristic spiral structure of ORFV. Scale bars: 50 nm. **(D)** PCR amplification of ORFV 011 (269 bp in size), ORFV 020 (381 bp in size), and ORFV 059 gene (256 bp in size) of ORFV-CL24 strain in passages 2, 4, and 6 (P2, P4, and P6), compared with uninfected OFTu cells (Control) and ORFV-SY17-infected cells (Positive control). **(E)** A specific band was detected for ORFV protein at a molecular weight of approximately 50 kDa in serially passaged viruses (P4 and P6) in OFTu cells. GAPDH (37 kDa) was used as an internal loading control.

Virus isolation was performed using the scab specimens of affected sheep. In brief, the homogenized suspensions of the collected scab specimens were centrifuged, filtered, and then inoculated onto confluent primary ovine fetal turbinate cells (OFTu) monolayers. As shown in Figure 3B, after five blind passages in OFTu cells, the obvious cytopathic effects (CPEs) that consisted of enlarged and rounded cells, followed by cell shrinkage and detachment, were observed in OFTu cells inoculated with tissue homogenates. As ORFV causes progressive damage, it spreads from initially infected cells to adjacent cells, culminating in complete monolayer destruction within 72–96 h. However, no CPE was observed in the control cells (Figure 3A). The supernatant of the CPE-positive OFTu cell culture was used as a material for subsequent PCR detection. The ORFV 011, ORFV 020, and ORFV 059 genes were amplified from serially passaged viruses, including passages 2, 4, and 6. The amplified products were analyzed via agarose gel electrophoresis, which showed the approximate expected DNA banding pattern on a gel (Figure 3D). Further transmission electron microscopy (TEM) observation revealed enveloped, ovoid-shaped viral particles with a diameter of 150–300 nm, which were consistent in structure with poxviruses. Moreover, the characteristic criss-cross pattern of spiral filament wrapping around the viral membrane’s tubular scaffolding was observed (Figure 3C). Moreover, specific immunoreactive bands were detected at 50 kDa corresponding to ORFV protein by Western blotting (Figure 3E).

### Virus infectivity assay

**Figure 4:**
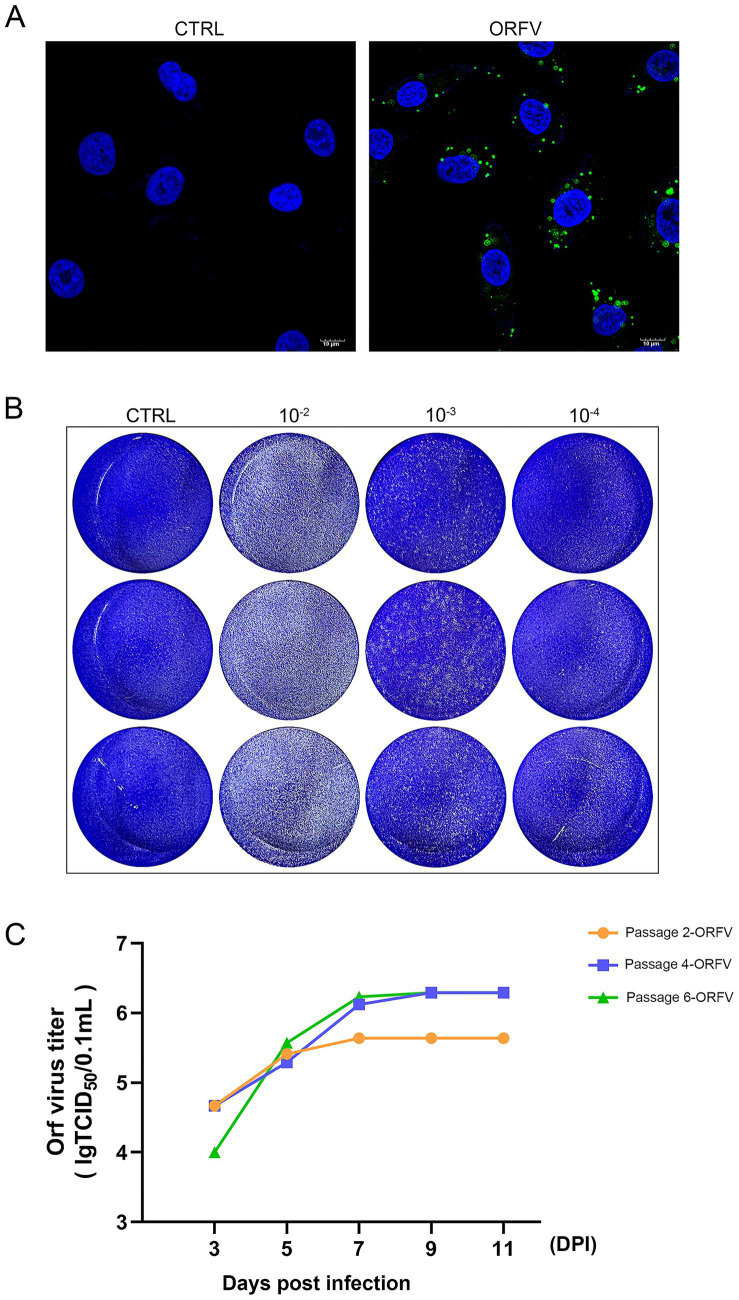
Viral infectivity assay. **(A)** IFA revealed that the ORFV-CL24 strain (green) was accumulated in the perinuclear region of infected OFTu cells, while no distinct green fluorescence was observed in the control cells. The cell nucleus was stained with DAPI (blue). **(B)** The infectious virus titer of the culture supernatant of OFTu cells following infection with ORFV-CL24 strain (passage 6) was subjected to viral plaque assay on confluent monolayers of OFTu cells. Plaque morphology of ORFV-CL24 strain at different dilutions using the direct agarose overlay plaque assay. Viral titer was calculated as: PFU/mL=(plaque number)×(dilution factor)^(−1)×(inoculum volume)^(−1) . **(C)** Viral replication kinetics of ORFV-CL24 strain *in vitro*. The infected cultures for serially passaged viruses, including the 2nd passage (yellow), the 4th (blue), and the 6th (green), were collected at the time when CPE reached 75%. Viral titers were calculated as log10 tissue culture infective dose 50 (TCID_50_)/0.1 mL, which were 10^5.64^, 10^6.29^, and 10^6.23^, respectively.

We measured viral infectivity with an assay that employed a fluorescent immunostaining technique to detect the distribution of ORFV-CL24 particles in infected OFTu cells. Immunofluorescence assay (IFA) revealed that viral antigens (green) were aggregated in the perinuclear region of infected OFTu cells, while no fluorescence was observed in the mock-infected cells (Figure 4A). The viral titers (TCID_50_/0.1 mL) of the harvested supernatants in passages 2, 4, and 6 (P2, P4, and P6) were determined in OFTu cells and calculated using the Reed–Muench method. As shown in Figure 4C, the TCID_50_ titers for serially passaged viruses (the 2nd, 4th, and 6th passages) were 10^5.64^, 10^6.29^, and 10^6.23^, respectively, indicating that the ORFV-CL24 strain was stable in viral replication. We further assessed the infectivity of ORFV-CL24 particles using a plaque assay. Cytopathic effect with virus plaques was clearly visible across all tested culture passages, thus confirming that no obvious decrease in infectivity was observed even throughout the serial passages (Figure 4B and Supplementary Figure 1).

### Genome structure of ORFV

**Figure 5:**
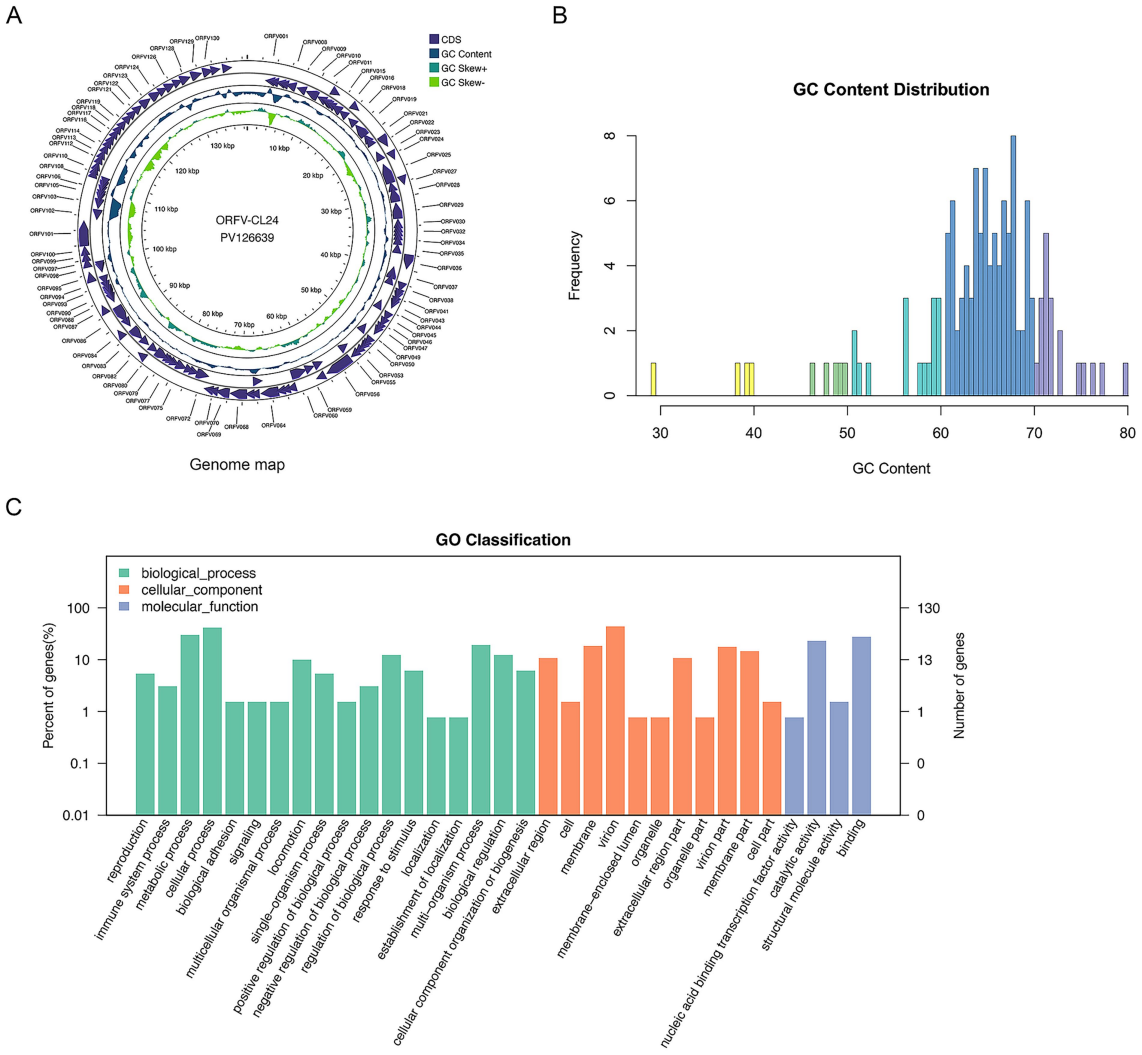
The annotation and visualization of ORFV-CL24 genome. **(A)** Circular map of genomic features of ORFV-CL24 strain. The map illustrated the characteristic genomic structure of ORFV-CL24 strain, where the outer ring represented the annotated ORFs, the purple tracks represented protein-coding sequences (CDS), the blue ring, uneven circle in the middle represented the GC content distribution along the genome, the green rings represented GC skew (±) patterns, and the central scale indicated the genomic position (kbp). The functional classification of the genes was shown in the upper right corner. **(B)** The GC content analysis showed a predominant 60–70% composition with underrepresentation at 30–60% and 70–80%, indicating significant genomic bias. **(C)** Gene Ontology (GO) functional classification of ORFV-CL24. The distribution of 131 ORFs among the three major GO categories (Biological Process, Cellular Component, and Molecular Function) was shown. The vertical axis represented the specific level-2 GO terms within each category. The horizontal axis represented specific biological processes or functions. The left vertical axis indicated the percentage of genes per term relative to the total annotated genes, and the right vertical axis showed the number of genes annotated for each term.

**Figure 6:**
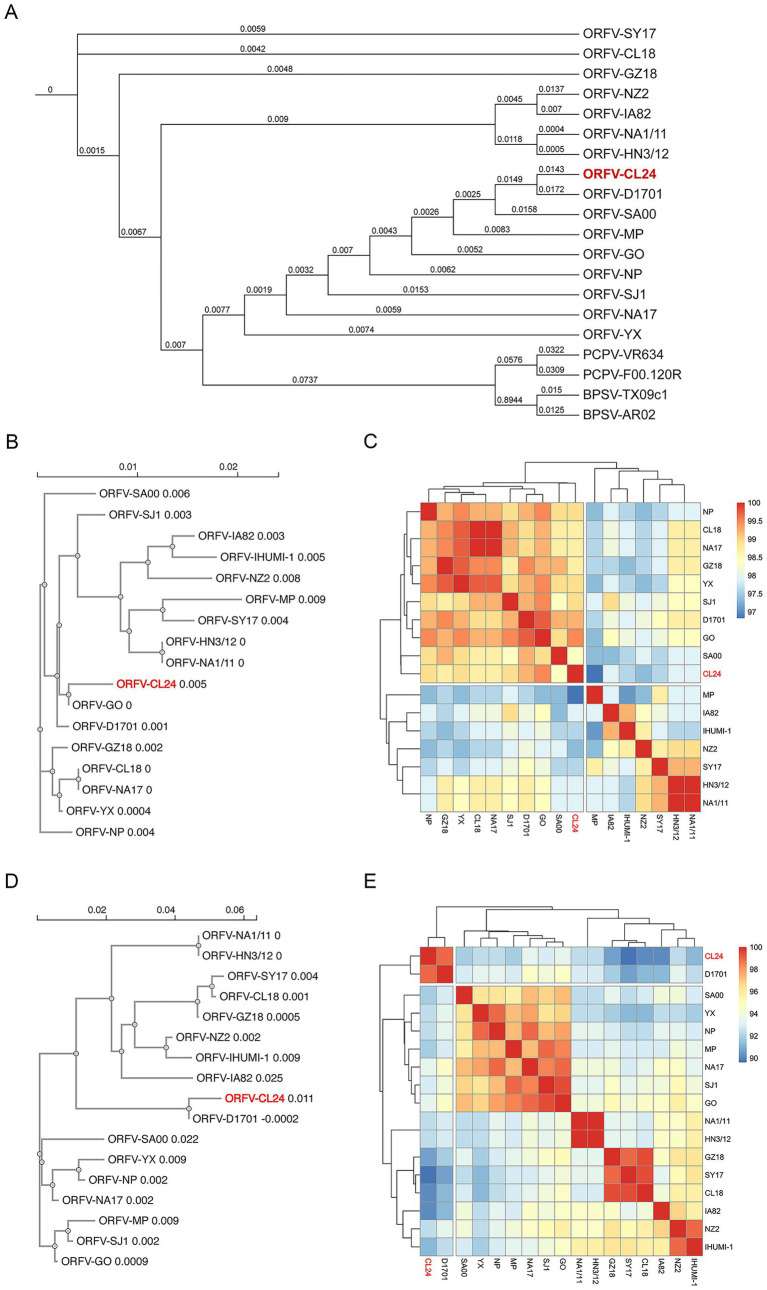
Phylogenetic analysis of parapoxviruses (PPVs). **(A)** Maximum likelihood phylogenetic tree constructed from whole genome sequences using MAFFT for alignment, TrimAI for trimming, and IQ-TREE 2 (TVM + F + I + R2 model) for tree reconstruction, with bootstrap values (1,000 replicates) shown at branch nodes to indicate evolutionary relationships. **(B,D)** Neighbor-joining phylogenetic trees of ORFV 011 **(B)** and ORFV 020 **(D)** gene sequences generated using Clustal Omega, demonstrating strain-specific clustering patterns. **(C,E)** Heatmap visualization of pairwise amino acid sequence differences for ORFV 011 **(C)** and ORFV 020 **(E)** based on multiple sequence alignments performed with Clustal Omega and analyzed using R language packages, with color gradients representing degrees of sequence conservation (red: high conservation; blue: high divergence).

**Figure 7:**
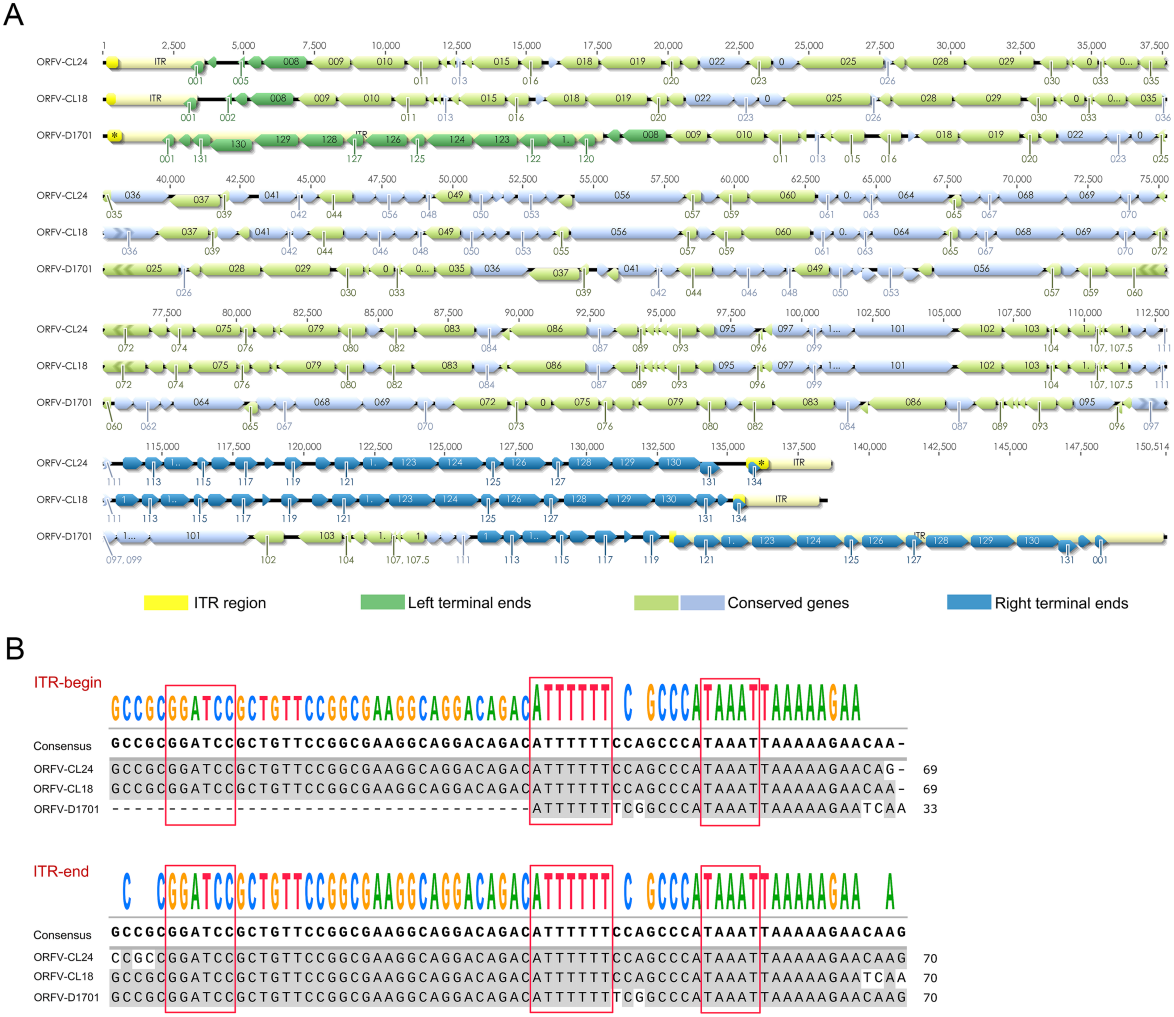
Genomic structure and ITR alignment analysis of ORFV-CL24 strain. **(A)** Comparative genomic map for ORFV-CL24 with ORFV reference strains (including ORFV-CL18 and D1701). The predicted ORFs are shown by colored arrows. The ITR regions at both genomic termini were depicted as yellow segments. The ORFs at the variable terminal region were shown in green, while those at the right terminal variable region were indicated in blue. Conserved central genomic regions were color-coded in light green (reverse orientation) and light blue (forward orientation). **(B)** Multiple sequence alignment of ITR regions of ORFV-CL24 and ORFV reference strains, highlighting conserved genomic features with red boxes marking the BamHI restriction site (GGATCC) and telomere resolution sequence (ATTTTTT-N8-TAAAT).

The complete genome sequence of the ORFV-CL24 strain obtained by gene sequencing, assembly, and annotation was 138,500 bp with 131 open reading frames (ORFs) (Supplementary Table 1) and was submitted to GenBank (accession number: PV126639). The ORFV-CL24 strain has an overall GC content of 63.3%, predominantly ranging between 60 and 70% across individual genes. The characteristic terminal GC-skew pattern was a hallmark feature of parapoxviruses (Figures 5A,B). Furthermore, Gene Ontology (GO) analysis revealed significant enrichment of genes associated with core biological processes, including metabolism, cellular organization, catalytic activity, and molecular binding. However, those involved in advanced regulatory functions were underrepresented; this might be beneficial in explaining the virus’s dependence on host machinery for complex functions (Figure 5C). Based on the genome-wide sequence alignment of diverse strains listed in Table 1, the nucleotide sequence homology between CL24 and the 16 representative ORFV strains ranged from 93.6 to 97.0% (Supplementary Figure 2). The ORFV-CL24 strain showed the highest similarity (97.0% nucleotide identity) to D1701, and ORFV-NP had the lowest similarity (93.6% nucleotide identity). Phylogenetic analysis based on the ORFV complete genome sequence revealed the close relationship of ORFV-CL24 to the D1701 strain (Figure 6A). Thus, the evolutionarily related ORFV-D1701 strain and the geographically co-circulating ORFV-CL18 strain were selected as reference strains to perform further comparisons with the ORFV-CL24 strain. Through genomic data visualization and structural annotation analyses, and comparative genomic analysis revealed that the ORFV-CL24 genomic structure included a highly conserved central region (ORFs 009-120) containing genes that identified as essential for viral replication, flanked by variable terminal regions (ORFs 001-008 and ORFs 121-134) harboring these viral proteins that related to viral-host interactions, and bounded by 3,264 bp inverted terminal repeats (ITRs) that were critical for genome stability (Figure 7A). Furthermore, the potential conserved functional elements, including BamHI restriction sites (GGATCC) and telomere resolution motifs (ATTTTTT-N8-TAAAT), were identified by detailed examinations of ITR regions. Additionally, an exceptional 18 kbp ITR was observed in ORFV-D1701 due to recombination between nonhomologous sequences (Figure 7B). Together, these findings provided a new genomic blueprint of the ORFV-CL24 strain, which would yield valuable insights into the strain-specific variations in the critical regions, particularly in the ITR regions that mediated viral replication and evolutionary adaptation.

**Table 1:** Twenty-one fully sequenced PPVs used in this study.

PPV species	Isolate	Host	Country	GeneBank accession	Predicted genes	Genome size (bp)	ITR size (bp)	GC content (%)	References
ORFV	CL24	Sheep	China	PV126639	131	138,500	3,264	63.3	This study
ORFV	CL18	Sheep	China	MN648219	131	138,495	3,481	63.8	Zhou et al. (2022)
ORFV	GZ18	Sheep	China	MN648218	131	138,446	3,469	63.9	Zhou et al. (2022)
ORFV	SY17	Sheep	China	MG712417	131	140,413	4,267	63.8	Zhong et al. (2019)
ORFV	IA82	Sheep	USA	AY386263	130	137,241	3,092	64.3	Delhon et al. (2004)
ORFV	D1701*	Sheep	Germany	HM133903	259	134,038	18,000	63.7	Cottone et al. (1998)
ORFV	NA1/11	Sheep	China	KF234407	132	137,080	3,020	63.6	Li et al. (2015)
ORFV	HN3/12	Sheep	China	KY053526	132	136,643	2,794	63.7	Chen et al. (2017)
ORFV	NZ2	Sheep	New Zealand	DQ184476	132	137,820	3,389	64.3	Mercer et al. (2006)
ORFV	NA17	Goat	China	MG674916	132	139,287	3,974	63.7	Zhong et al. (2019)
ORFV	SJ1	Goat	China	KP010356	129	139,112	4,153	63.6	Chi et al. (2015)
ORFV	YX	Goat	China	KP010353	132	138,231	3,446	63.8	Chi et al. (2015)
ORFV	NP	Goat	China	KP010355	124	132,111	2,426	63.8	Chi et al. (2015)
ORFV	GO	Goat	China	KP010354	132	139,866	3,964	63.6	Chi et al. (2015)
ORFV	MP	Goat	India	MT332357	132	139,807	3,910	63.7	Sahu et al. (2022)
ORFV	SA00	Goat	USA	AY386264	130	139,962	3,936	63.4	Delhon et al. (2004)
ORFV	IHUMI-1	Human	France	LR594616	126	132,823		64.1	Andreani et al. (2019)
PCPV	VR634	Human	Finland	GQ329670	134	145,289	14,909	65.0	Hautaniemi et al. (2010)
PCPV	F00.120R	Reindeer	Finland	GQ329669	131	133,169	2064	64.1	Hautaniemi et al. (2010)
BPSV	TX09c1	Cow	USA	KM875472	129	135,072	1,415	64.4	Huang et al. (2015)
BPSV	AR02	Calf	USA	AY386265	131	134,431	1,161	64.5	Delhon et al. (2004)

* The ORFV-D1701 strain has an exceptionally large ITR due to recombination.

### Phylogenetic analysis

To determine the origin and evolutionary relationships of ORFV-CL24 to other representative viruses within the *Parapoxvirus* genus, we conducted a comprehensive phylogenetic analysis based on the whole-genome data by employing a rigorous computational pipeline. The detailed description of the analytical workflow included the multiple sequence alignment listed in Table 1 using MAFFT’s FFT-NS-2 algorithm, followed by stringent quality filtering with TrimAI to retain phylogenetically informative sites (<80% gaps, >60% sequence consistency). Maximum likelihood phylogenetic reconstruction in IQ-TREE 2 with 1,000 replicates each of SH-aLRT and ultrafast bootstrap analysis, providing robust statistical support for all topological features. Genomic analysis revealed the ORFV-CL24 strain exhibited 97% sequence identity with its closest relative, ORFV-D1701, and 95.1% identity with ORFV-SJ1 (Supplementary Figure 2). Phylogenetic reconstruction demonstrated strong monophyletic clustering of all ORFV strains, indicating their shared evolutionary origin. However, ORFV-CL24 clustered within a subclade with ORFV-D1701, showing close genetic relatedness while maintaining slight evolutionary divergence from other subgroups among ORFV, as evidenced by its unique branching pattern. This subclade differentiation suggested the potential strain-specific evolutionary adaptations in ORFV-CL24. Consistent with established taxonomy, PCPV and BPSV strains formed separate, well-supported clades with substantial genetic distance from ORFV strains (Figure 6A). These findings provided novel insights into the phylogenetic relationships and genetic diversity patterns within the ORFV lineage, highlighting the distinct evolutionary trajectory of the newly characterized ORFV-CL24 strain.

Moreover, phylogenetic analysis based on ORFV B2L (ORFV 011) and E3L (ORFV 020) genes provided detailed insights into ORFV genetic diversity and evolutionary patterns. Our integrated bioinformatics pipeline, incorporating Clustal Omega for multiple sequence alignment and neighbor-joining phylogenetic reconstruction (1,000 bootstrap replicates), revealed distinct molecular signatures in ORFV-CL24. Analysis of ORFV 011 revealed the characteristic amino acid substitutions (V80I, V101I; Supplementary Figure 3A) and demonstrated a close evolutionary relationship among ORFV-CL24, D1701, and GO, suggesting a common origin. Furthermore, these strains clustered into a tightly related sub-lineage, while ORFV-HN3/12 and NA1/11 formed another distinct but equally conserved sub-lineage (branch length = 0, reflecting genetic distance). Both sub-lineages were clearly separated from more divergent strains such as SY17 (branch length = 0.004) and MP (branch length = 0.009), demonstrating the genetic diversity of ORFV (Figure 6B). Further analysis of ORFV 020 showed that ORFV-CL24 (branch length = 0.011) and D1701 (branch length = −0.0002) comprised a recently diverged cluster, and strains GZ18, CL18, and NZ2 form a cluster with >70% bootstrap support, which was sister to the cluster containing IA82 and SA00. Notably, despite their phylogenetic relatedness, the distinct position of ORFV-CL24 from D1701 was further supported by specific ORFV 020 mutations (S14Q, R123K; Supplementary Figure 3B), suggesting potentially evolutionary dynamics and host adaptation mechanisms. Comparative heatmap analysis of ORFV 011 (Figure 6C) and ORFV 020 (Figure 6E) genes confirmed the genetic divergence of the ORFV-CL24 strain, showing consistent patterns that align with phylogenetic data. These findings demonstrated that the ORFV-CL24 strain possessed a unique evolutionary mechanism and suggested potential functional differences in viral proteins that may impact biological characteristics, meriting further molecular investigation.

## Discussion

Orf is a globally distributed disease with high transmissibility, caused by ORFV, predominantly endemic in sheep, goats, and other ruminant populations, which could be transmitted through contact with infected individuals (Lawan et al., 2021; Abu Ghazaleh et al., 2023). ORFV causes seasonal outbreaks in flocks, usually during the spring and winter seasons. Although ORFV typically exhibits low mortality rates in adult animals, it has displayed flock mortality rates of up to 90% in lambs, causing economic impacts on livestock industries through loss of milk production, poor meat quality, and decreased yield (Onyango et al., 2014; Bala et al., 2018). In addition, humans can be infected by direct or indirect contact with infected animals (Zuelgaray et al., 2018), most often manifesting as lesions on hands and fingers; occasionally, facial involvement is noted, including nasal, labial, and periocular regions, indicating possibly increased zoonotic potential (Freeman et al., 1984; Bodnar et al., 1999; Aslan Kayıran et al., 2018). Livestock vaccination offers a feasible control measure in effectively reducing ORFV transmission to sheep (Bukar et al., 2021). However, the currently available conventional vaccines typically lose efficacy due to the viral genetic variability, possible cross-species transmission, and the unique immune evasion mechanism, and so on (Haig and McInnes, 2002; Wang et al., 2019). Although ongoing research focuses on developing new vaccines and immunotherapies, little is known about the distribution, prevalence, and severely impacted areas in China. Relatively few virus isolates have been recovered and sequenced, and the pathogenesis of the virus remains poorly understood. In the present study, we successfully isolated and characterized a novel ORFV strain (designated CL24, GenBank accession: PV126639) from a naturally infected sheep using transmission electron microscopy, cytopathic effect observation, PCR, immunofluorescence assay, and plaque assay.

To gain deeper insights into viral gene function, genetic diversity, and evolutionary relationships of the newly isolated strain, the complete genome sequence of the ORFV-CL24 strain was sequenced and analyzed using the Illumina NovaSeq 6,000 platform with a paired-end read size of 300 bp. As expected, the central coding regions (ORFs 009-120) of the ORFV-CL24 genome exhibit high sequence identity and can be highly conserved in structure, which contain genes that have been identified as essential for viral replication. Further analysis of the genome sequence showed that the variable terminal regions (ORFs 001–008 and ORFs 121-134) harboring these viral proteins that related to virulence, immune regulation and viral-host interactions, and bounded by 3,264 bp inverted terminal repeats (ITRs) at both termini that were critical for genome stability, which was recognized as the typical genomic organization and structural characteristic of the member of the genus *Parapoxvirus* (Fraser et al., 1990). It was worth noting that the potential characteristic structural motif found in viral ITRs, including a BamHI restriction site (5′-GGATCC-3′) and the highly conserved telomere resolution motif (5′-ATTTTTT-N8-TAAAT-3′), was shown to be highly conserved among different ORFV strains. Although it was likely to be associated with viral pathogenesis, the exact mechanism was mostly poorly understood (Merchlinsky, 1990). Thus, studies are needed to further investigate this hypothesis. Our research not only provides a high-quality comparative genomic resource but also will deepen the understanding of parapoxvirus genetic evolution.

Currently, the evolutionary relationships of ORFV remain unclear due to a lack of sufficient molecular data. To further reveal the origin and evolutionary pattern of ORFV-CL24 strain, phylogenetic analysis based on the 20 representative PPVs whole genome sequences was subsequently performed using MAFFT for alignment, TrimAI for trimming, and IQ-TREE 2 for tree reconstruction. As shown in Figure 6A, the genome of the ORFV-CL24 strain was closely related to the highly attenuated vaccine strain D1701, which was known for extensive genomic deletions and rearrangements, particularly a massive ~18 kbp ITR. So, it seems genetically impossible for ORFV-CL24 to be derived from ORFV-D1701 by recombining the ITR sequence due to the large size of the ITR region. Thus, we speculated that the ORFV-CL24 strain might share a putative common ancestor with ORFV-D1701 or be derived from D1701 attenuated precursors. To exclude the possibility that the high similarity might be due to the analysis focusing on the conserved central core of the genome, thus masking significant differences in the variable termini that dictate virulence, we conducted a detailed alignment analysis of ORFV-CL24 and ORFV-D1701 genomes. As shown in Supplementary Figures 4A–D, ORFV-CL24 shared a high homology of 97.3% with ORFV-D1701 in the absence of the terminal variable region, which was consistent with the alignment results based on the PPV’s whole genomes (Supplementary Figure 2). Meanwhile, the comparisons based on the terminal ITR regions were made between ORFV-CL24 and ORFV-D1701. It was found that the sequence homology of ITRs was less than 20% (Supplementary Figures 4E,F), which might be the main reason contributing most to the virulence phenotype. Considering that the evolutionary analysis of the large genome size prioritizes matching the highly conserved core genome, thus ignoring the differences in small fragments at terminals, which led to such a paradox that having a close relationship and the difference in virulence phenotype. On the other hand, the sequence divergence between them also indicated there was a potential evolutionary pattern marked by both remarkable conservation and selective diversification. Thus, we speculated that ORFV could employ a dual evolutionary strategy, maintaining strict conservation in functionally critical regions like ITRs while permitting adaptive diversification in host-interaction genes. The sequence or structural variability in function might be relevant to the evolution of specific genes, e.g., ORFV 011 gene possessing V80I and V101I substitutions, while ORFV 020 gene harboring S14Q and R123K mutations. The identified amino acid substitutions, particularly in immunomodulatory genes, likely represent molecular adaptations with potential implications for host range and pathogenicity, indicating that potential strain-specific evolutionary adaptations might have existed in ORFV-CL24. Furthermore, phylogenetic trees were constructed based on individual genes (ORFV 011 and ORFV 020 genes) sequences (Figures 6B,D). This study not only revealed the evolutionary traits and potential genetics but also provided new insight into the origin and evolution of the ORFV-CL24 strain.

In conclusion, the comprehensive genomic characterization of ORFV-CL24 has been explored and yielded important insights into the genetic variation and evolutionary divergence of ORFV circulating in Northeast China. Furthermore, these findings provided valuable data for further research on revealing the immune regulatory mechanism, optimizing control strategies, thus reducing economic losses in sheep or goat industries, and zoonotic transmission risks.

## Data Availability

The datasets presented in this study can be found in online repositories. The names of the repository/repositories and accession number(s) can be found in the article/Supplementary material.

## Footnotes

^1^ https://www.ebi.ac.uk/jdispatcher/msa/clustalo

^2^ https://www.snapgene.com

^3^ https://proksee.ca

^4^ https://www.geneious.com

## Supplementary material

SUPPLEMENTARY FIGURE 1 Viral plaque assay. **(A,B)** The infectious virus titer of the culture supernatant of OFTu cells following infected respectively with ORFV-CL24 passage 2 **(A)** and passage 4 **(B)** was subjected to viral plaque assay on confluent monolayers of OFTu cells. Plaque morphology of ORFV-CL24 strain using the direct agarose overlay plaque assay. Viral titer was calculated as plaque-forming units (PFU) based on a mathematical formula: PFU/mL=(plaque number)×(dilution factor)^(−1)×(inoculum volume)^(−1) .

SUPPLEMENTARY FIGURE 2 Multiple sequence alignment (MSA) of ORFV complete genome listed in Table 1.

SUPPLEMENTARY FIGURE 3 MSA of ORFV 011 and ORFV 020 listed in Table 1. **(A,B)** MSA of ORFV 011 **(A)** and MSA of ORFV 020 **(B)** were performed with Clustal Omega and visualized using Jalview.

SUPPLEMENTARY FIGURE 4 Pairwise alignment between ORFV-CL24 and ORFV-D1701. **(A)** Alignment of the complete genomes of ORFV-CL24 and ORFV-D1701 was performed using MAFFT. **(B)** Alignment of the core genome (excluding ITR regions) of ORFV-CL24 and ORFV-D1701 was performed using MAFFT. **(C,D)** The percent identity of genomic nucleotides between ORFV-CL24 and ORFV-D1701 for the whole genome **(C)** and the core genome **(D)**. **(E)** The ITR sequences at the left terminus of ORFV-CL24 was aligned with that of ORFV-D1701 using MAFFT. **(F)** The ITR sequence at the right terminus of ORFV-CL24 was aligned with that of ORFV-D1701 using MAFFT.

SUPPLEMENTARY TABLE 1 Genome annotations of ORFV-CL24 strain. The complete genome sequence of ORFV-CL24 strain obtained by gene sequencing and assembly was 138500 bp with 131 open reading frames (ORFs), which were annotated using Gene Ontology (GO) analysis.
