# Supplementary figures and images for: The identification and genetic characteristics of the Orf virus strain (ORFV-CL24) isolated from Jilin province, China
Source: Front Microbiol. 2025 Sep 24;16:1658326. doi: 10.3389/fmicb.2025.1658326 (PMC12504275; doi:10.3389/fmicb.2025.1658326)

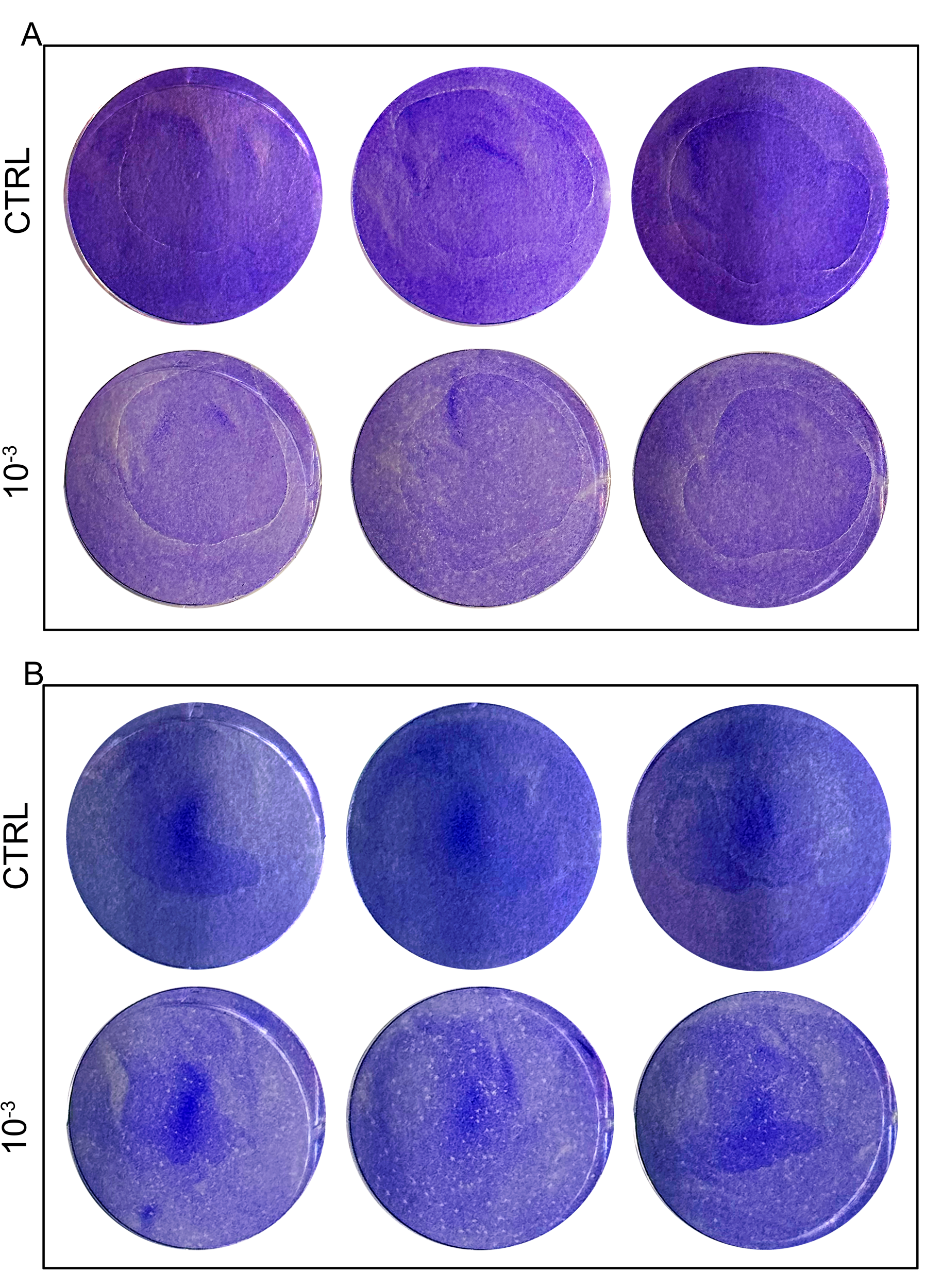

Supplement: SUPPLEMENTARY FIGURE 1 — Viral plaque assay. (A,B) The infectious virus titer of the culture supernatant of OFTu cells following infected respectively with ORFV-CL24 passage 2 (A) and passage 4 (B) was subjected to viral plaque assay on confluent monolayers of OFTu cells. Plaque morphology of ORFV-CL24 strain using the direct agarose overlay plaque assay. Viral titer was calculated as plaque-forming units (PFU) based on a mathematical formula: PFU/mL=(plaque number)×(dilution factor)^(−1)×(inoculum volume)^(−1) . [file Image_1.tiff]

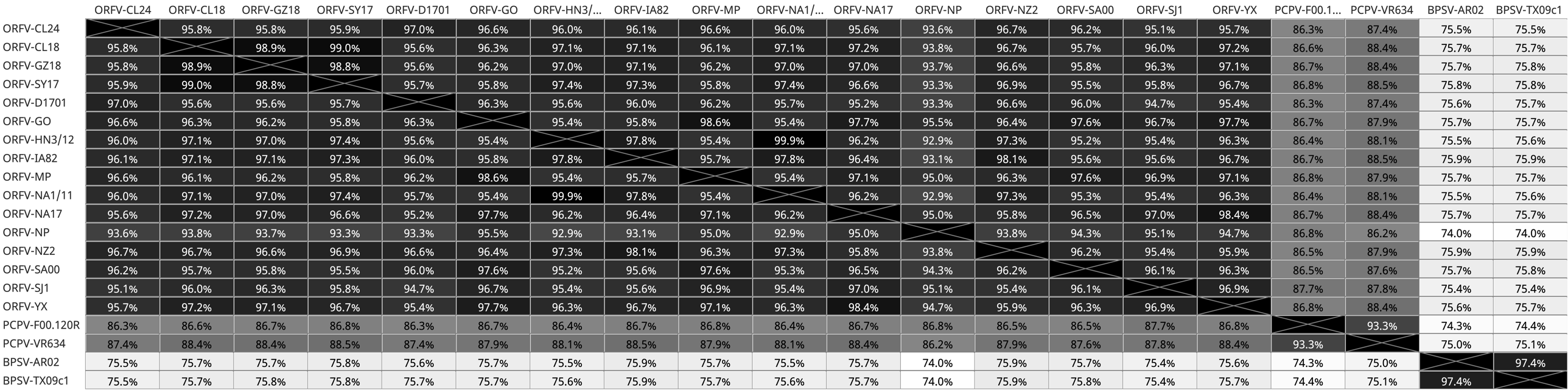

Supplement: SUPPLEMENTARY FIGURE 2 — Multiple sequence alignment (MSA) of ORFV complete genome listed in Table 1. [file Image_2.tif]

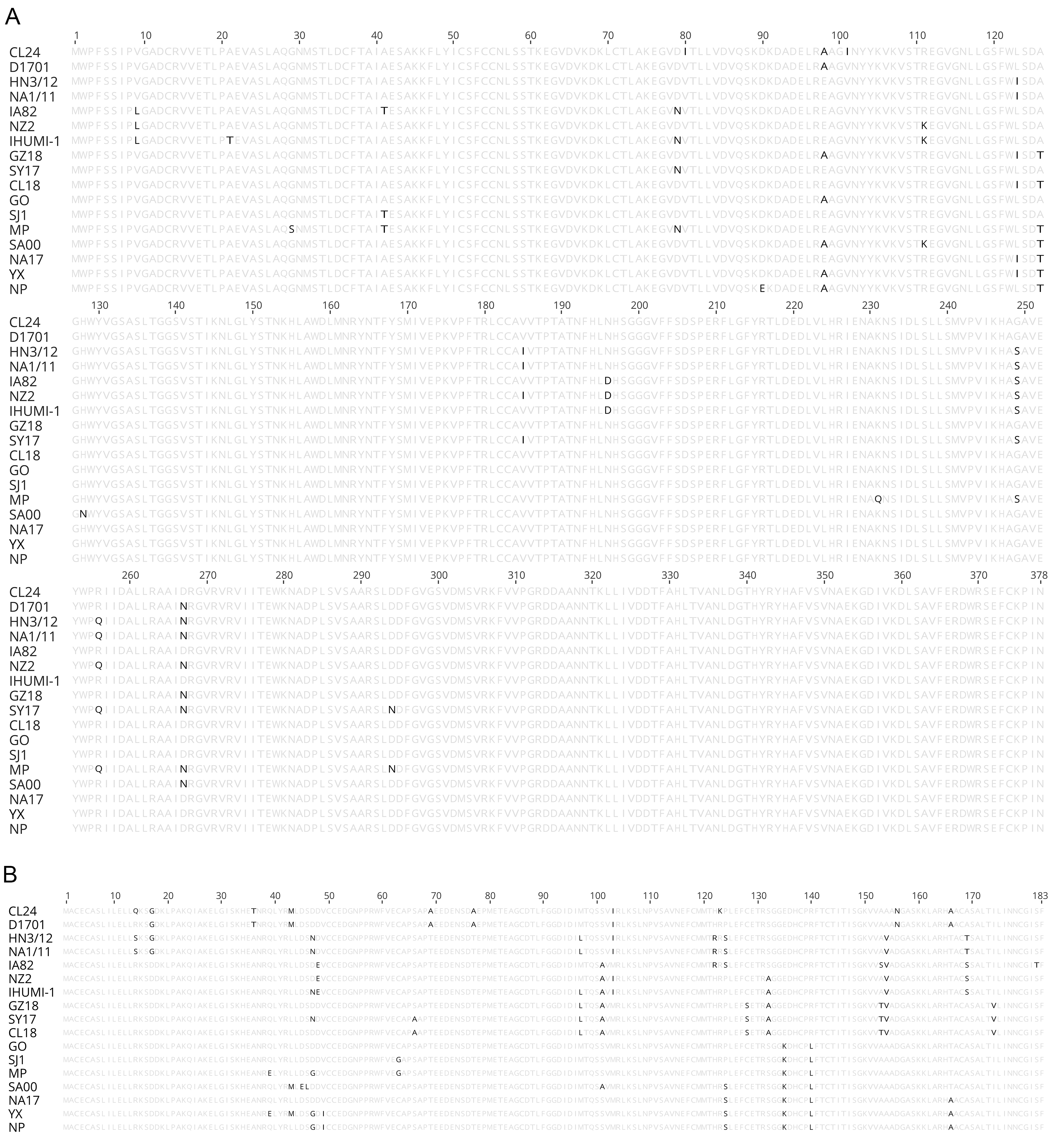

Supplement: SUPPLEMENTARY FIGURE 3 — MSA of ORFV 011 and ORFV 020 listed in Table 1. (A,B) MSA of ORFV 011 (A) and MSA of ORFV 020 (B) were performed with Clustal Omega and visualized using Jalview. [file Image_3.tif]

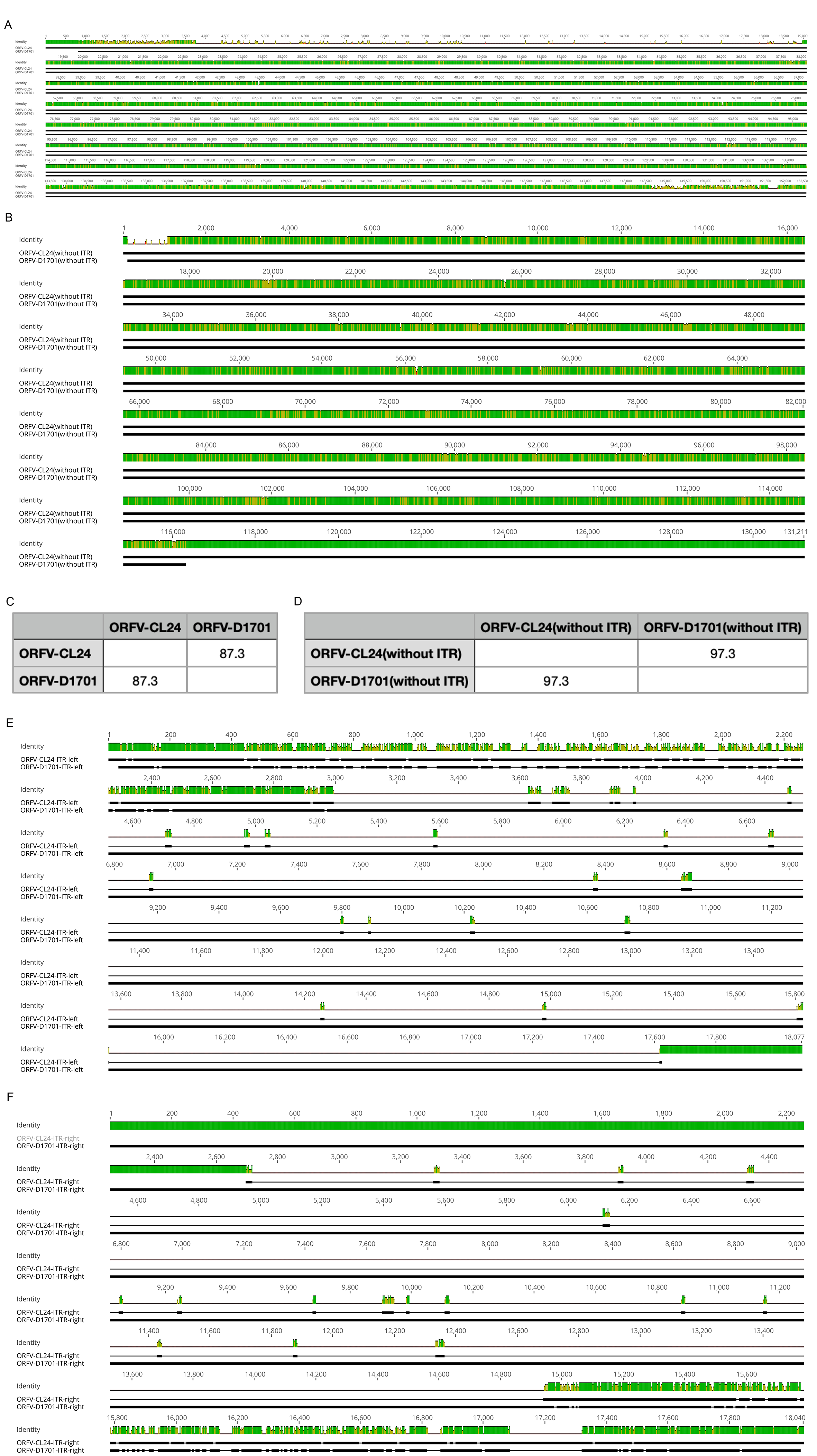

Supplement: SUPPLEMENTARY FIGURE 4 — Pairwise alignment between ORFV-CL24 and ORFV-D1701. (A) Alignment of the complete genomes of ORFV-CL24 and ORFV-D1701 was performed using MAFFT. (B) Alignment of the core genome (excluding ITR regions) of ORFV-CL24 and ORFV-D1701 was performed using MAFFT. (C,D) The percent identity of genomic nucleotides between ORFV-CL24 and ORFV-D1701 for the whole genome (C) and the core genome (D). (E) The ITR sequences at the left terminus of ORFV-CL24 was aligned with that of ORFV-D1701 using MAFFT. (F) The ITR sequence at the right terminus of ORFV-CL24 was aligned with that of ORFV-D1701 using MAFFT. [file Image_4.tif]

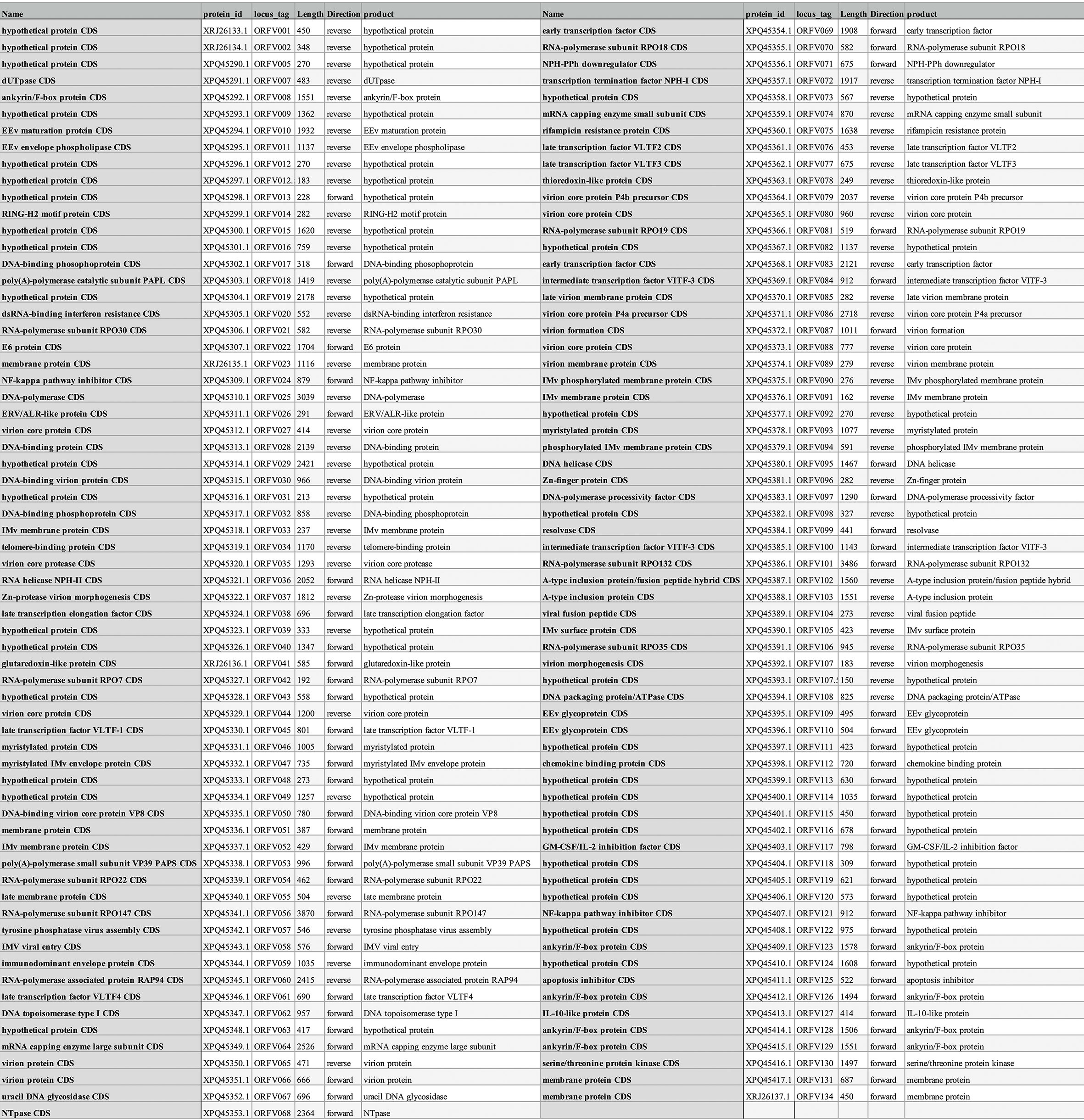

Supplement: SUPPLEMENTARY TABLE 1 — Genome annotations of ORFV-CL24 strain. The complete genome sequence of ORFV-CL24 strain obtained by gene sequencing and assembly was 138500 bp with 131 open reading frames (ORFs), which were annotated using Gene Ontology (GO) analysis. [file Image_5.tif]
